# In Vivo Validation of Diffuse Optical Imaging with a Dual-Direction Measuring Module of Parallel-Plate Architecture for Breast Tumor Detection

**DOI:** 10.3390/biomedicines10051040

**Published:** 2022-04-30

**Authors:** Jhao-Ming Yu, Liang-Yu Chen, Min-Cheng Pan, Ya-Fen Hsu, Min-Chun Pan, Yi-Ling Lin, Sheng-Yih Sun, Chia-Cheng Chou

**Affiliations:** 1 Department of Mechanical Engineering, National Central University, Taoyuan City 320, Taiwan; jming.yu@ude-corp.com (J.-M.Y.); liang_yu@alumni.ncu.edu.tw (L.-Y.C.); lynn_yl_lin@wistron.com (Y.-L.L.); 2 Department of Electronic Engineering, Tungnan University, New Taipei City 222, Taiwan; m2pan@mail.tnu.edu.tw; 3 Department of Surgery, Landseed International Hospital, Taoyuan City 324609, Taiwan; hsuyf@landseed.com.tw; 4 Department of Radiology, Tao-Yuan General Hospital, No.1492, Zhongshan Rd., Taoyuan City 330, Taiwan; sysun@mail.tygh.gov.tw; 5 Department of Surgery, Tao-Yuan General Hospital, No.1492, Zhongshan Rd., Taoyuan City 330, Taiwan; surgery1@mail.tygh.gov.tw

**Keywords:** optical breast imaging system, diffuse optical imaging, dual-direction scanning, feature index

## Abstract

We demonstrate a working prototype of an optical breast imaging system involving parallel-plate architecture and a dual-direction scanning scheme designed in combination with a mammography machine; this system was validated in a pilot study to demonstrate its application in imaging healthy and malignant breasts in a clinical environment. The components and modules of the self-developed imaging system are demonstrated and explained, including its measuring architecture, scanning mechanism, and system calibration, and the reconstruction algorithm is presented. Additionally, the evaluation of feature indices that succinctly demonstrate the corresponding transmission measurements may provide insight into the existence of malignant tissue. Moreover, five cases are presented including one subject without disease (a control measure), one benign case, one suspected case, one invasive ductal carcinoma, and one positive case without follow-up treatment. A region-of-interest analysis demonstrated significant differences in absorption between healthy and malignant breasts, revealing the average contrast between the abnormalities and background tissue to exceed 1.4. Except for ringing artifacts, the average scattering property of the structure densities was 0.65–0.85 mm^−1^.

## 1. Introduction

The most commonly used biomedical imaging modalities for breast cancer diagnosis, such as X-ray mammography and sonography, can acquire structural information about breast tissue. However, these modalities are still limited when used in imaging overlapping structures, resulting in false diagnoses; furthermore, X-ray mammography involves ionizing radiation. Therefore, alternative techniques such as breast magnetic resonance imaging (MRI) has been explored for breast tumor screening. Meanwhile, photoacoustic imaging [1,2] and diffuse optical tomography/imaging (DOT/DOI) using near-infrared (NIR) light are emerging techniques that have been able to provide physiological and pathological information for over two decades. Both the absorption and scattering distribution of the breast can be obtained by NIR DOT [3,4,5,6,7,8,9,10,11,12,13,14]. More specifically, the oxyhemoglobin, deoxyhemoglobin, water, and lipid concentrations in breast tissue can be evaluated, provided that spectral information is available [4,5,8,9,10,11,13,14]. Multimodality imaging techniques to enhance reconstructed images have been developed through employing prior structural information and combining other image modalities for better detection and diagnosis [5,6,7,10,13,14]. Following phantom validation, in vivo test and/or clinical trials are usually conducted to assess the potential for developed diffuse optical tomographical imaging systems.

Recently, the use and improvement of DOT techniques through using paraxial scanning in the reconstruction of optical-property images has been applied in exploring the effects of compressive loading [15,16], developing multispectral imaging algorithms [17,18], assessing the reliability of optical properties [19], and even implementing a multispectral DOT associated with a 3T MRI system [20]. In the first pilot study regarding the application of X-ray mammography, DOI Zang et al. [21] developed both hardware and data processing algorithms for a multimodality imaging system; this provided a clearer understanding of the relationship between the functional contrast and the structural one. Using separately acquired digital mammograms as structural priors, a cost-effective approach incorporating tomographic optical image reconstruction resulted in improved spatial details estimation in the optical images [22]; further, a mammogram-guided DOT reconstruction algorithm was extended to facilitate the recovery of breast tumors, and a series of simulation-based investigations to evaluate the impact of lesion sizes and contrast levels, tissue background, mesh resolution, inaccurate priors, and regularization parameters was conducted [23]. It is worth noting that all these studies employed parallel-plate transmission geometry without considering the reflecting light from tissue or phantom.

We here first demonstrate a working prototype of an optical breast imaging system using a dual-direction scanning scheme of parallel-plate architecture. The scanning mechanism of this system was designed in combination with a mammography machine; thus, it can be operated while compressing a breast using two plates. With the help of prior mammogram examination, scanning sections can be selected to collect optical data for reconstructing diffuse optical images of tissue. Compared with transmission-only data, more information can be acquired from the dual-direction data collection design, which was realized with one pair of dual linear scanning arrays. Further, the intensity and phase information that provides insight into the existence of malignant tissue is characterized. This pilot study was intended to justify the proposed DOI scheme by imaging both healthy and malignant breasts in a clinical environment.

## 2. Instrumentation

This section provides an overview of the NIR DOI system with dual-direction transmission slabs designed at our laboratory, focusing on the following aspects: (1) the measuring architecture that describes light delivery and detection, and the optical fiber-phantom/patient interface, (2) the scanning mechanism that explains the dual-direction projection scheme, and (3) system calibration that elaborates on the experimental settings. This FD DOI system directly accounts for the effect of intensity and phase difference on optical-property reconstruction.

### 2.1. Measuring Architecture

**Figure 1:**
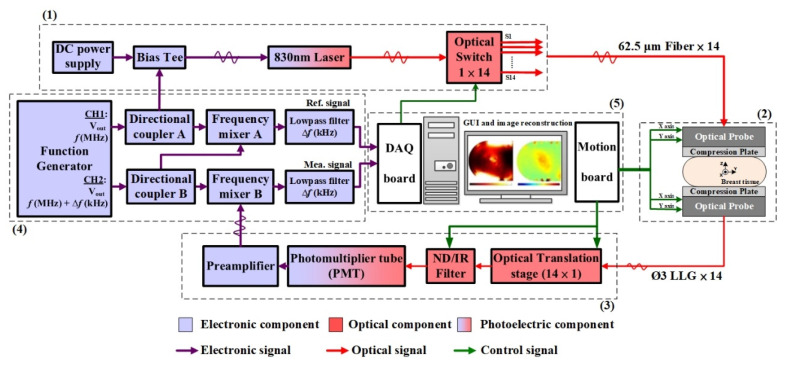
Measurement block diagram of the DOI system for compressed breast tissue, in which the optical probe includes an XY translation stage and fibers for source power and liquid light guides for tumor screening. For the dash-line blocks, (**1**): Light source module, (**2**): Scanning mechanism, (**3**): Detection module, (**4**) Signal modulation/demodulation, (**5**): Data acquiring and motion control module.

**Figure 2:**
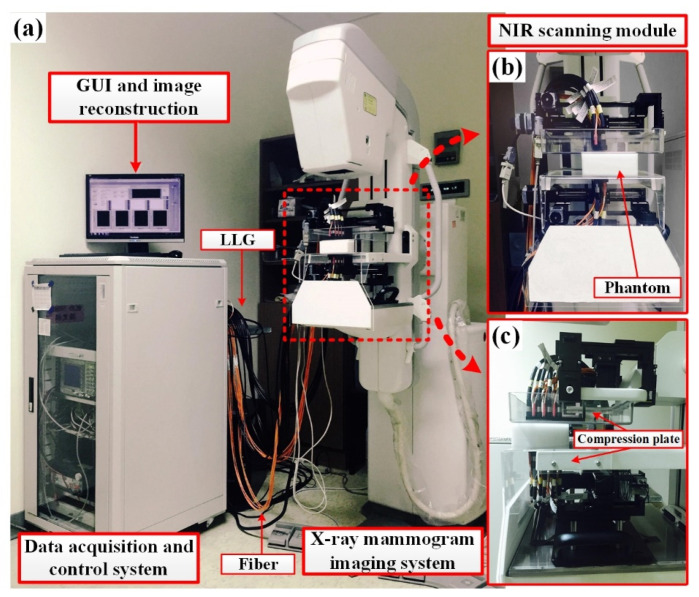
(**a**) Self-developed system in the clinical environment; (**b**) front view and (**c**) side view of the measuring device.

Figure 1 shows a block diagram of the tomographic DOI system. A picture of the system in a clinical environment is displayed in Figure 2a, with a close-up view of the measuring device provided in Figure 2b,c. The system was developed in the FD domain, and the craniocaudal (CC) view was adopted for optical data measurement. Currently, the system deploys a laser module (LDCU5/8202, Power Technology, Little Rock, AR, USA) with a wavelength of 830 nm, illumination of 3 mW, and 20 MHz alternating current, by which the NIR light source is guided into an optical switch (FOSW-1-16-N-62-L-2, Enaco, Taipei, Taiwan) using one input fiber and a bundle of 14 output fibers. These 15 fibers, each with a diameter of 1.2 mm, are composed of a pure silica core (62.5 µm) with a silicone clad, suitable for the transmission of wavelengths from 700 to 900 nm. The source light from the optical switch is delivered to the phantom/tissue through 14 extended fibers with the same 62.5-µm core; thus, each seven fibers are guided and mounted on the top and bottom slabs, respectively, composed of aluminum alloy (AL6061). Each of the fiber ends with a collimator makes contact with the optical plate. The fiber bundle is 1 m in length, extending from the instrument cart to the phantom/tissue interface. The efficiency of the optical switching is approximately 83%, yielding an average source power of 2.5 mW to the phantom/tissue surface.

**Figure 3:**
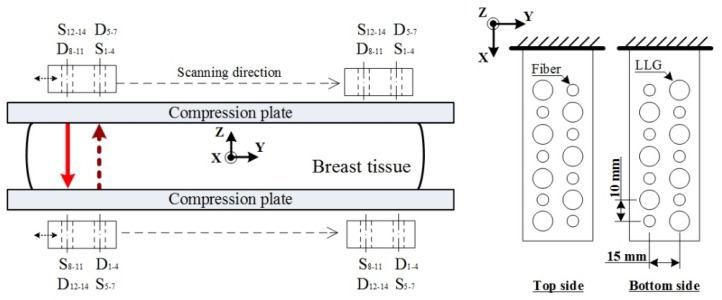
Schematic of a dual-direction projection scanning device.

For each of 7 source excitations in a single scanning section, the out-emitted light through the transmission or reflection paths is recorded from 4 (or 3) surface locations on two slabs, as shown in Figure 3, where the red solid arrow illustrates the NIR light from top (source) to low (detector), and vice versa. For both slabs, acquired signals are sensed by a photomultiplier tube (PMT, H11461-03, Hamamatsu, Japan) mounted on a translation stage (AL6061) and driven in sequence to 14 liquid light guides (LLGs, 77635, Newport, Irvine, USA) for the collection of out-emitted light. Subsequently, a neutral density filter (ND filter, OD 0.6–4, LAMBDA, Costa Mesa, USA) and an IR filter (830 ± 5 nm, 830FS10-12.5, Andover corp., Salem, USA) are set in front of the PMT to attenuate the received NIR power that fits the dynamic range of the PMT and additionally reduce the received light noise. A dedicated power supply (C7169, Hamamatsu, Japan) is used for PMT gain control. Varied gains are set for the PMT module by the dedicated power supply generating a driving voltage of 0.7–1.1 V to its control line; this enables the PMT to reliably sense optical signals in the range of nW to μW. Subsequently, the acquired photons are preamplified with a gain of 80. Intensity data are automatically acquired in series with a preprogrammed code for each detection.

A PC-based LabVIEW program running both a motion board and a multipurpose data acquisition board was coded to operate two scanning slabs, the translation stage, and the ND filter. It can also acquire data from the PMT with two analog channels and control the optical switch with one digital output line to change the source position. With a serially scanning and acquiring scheme, it takes approximately 90 s to measure one slice of the tested phantom/tissue for 146 detections [7S × 7D × 2 for transmission and (4S × 3D + 3S × 4D) × 2 for reflection] in total.

### 2.2. Scanning Mechanism

This NIR DOT scanning device was designed to be set up on a special plate compatible with an X-ray mammography apparatus (Senographe 2000D, GE, Boston, USA). Therefore, the device could be operated by compressing the breast with two plates to shorten the distance between sources and detectors, thus enhancing the signal-to-noise ratio during the measuring process; as in previous research, the parallel-plate scanning device was designed for this advantage [15,16,17,18,19,20]. In previous designs, however, the reconstruction image quality was restricted to the data obtained from single-direction information. Compared with a single-direction projection, our dual-direction scanning device was designed to contain one pair of dual linear scanning arrays (7 × 7) with a source-and-detector combination separately distributed on the top and bottom slabs (Figure 3). Details of this scanning device are explained as follows. Two scanning slabs are separately placed on the top and bottom plates; the slabs are made of optical polymethyl methacrylate (PMMA) (thickness: 2 mm) with a transmission rate of 95–98% and come into contact with the compressed phantom/breast. Each of the slabs machined from aluminum alloy is designed to possess a total of 14 ports: 7 fibers for source light and 7 LLGs for light detection. The separation between each fiber and LLG within each array is 10 mm, and that between two arrays is 15 mm. Both scanning slabs can move in the X or Y direction, controlled with a user-defined setting, such that the cross section of measurements can intersect the region of interest (ROI) in tissue. For the same scanning slice, one source array of the dual-direction scanning device is from one slab and the other is from the opposite slab; together, they acquire transmission and reflection data as shown in Figure 3. Compared with single-direction designs, this dual-direction measuring device can acquire relatively complete information to achieve better optical-property image quality [24].

**Figure 4:**
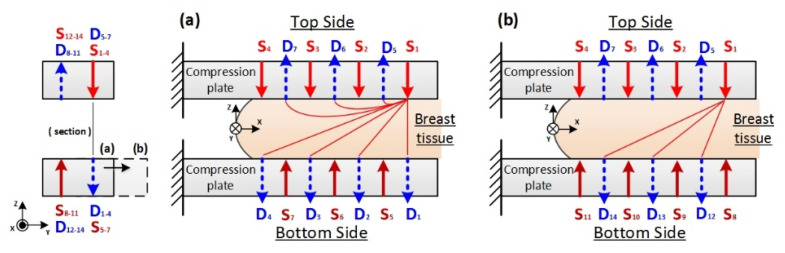
Dual-direction projection scheme with transmission and reflection information for an example of S1 from top to bottom, with (**a**) three reflections and four transmissions, and (**b**) three extra transmissions obtained by shifting the bottom slab, where red solid arrow and blue dash arrow denote NIR illumination and detection, respectively. The mark (**a**,**b**) on the left illustrate the bottom slab position for NIR illumination and detection shown in the subfigure (**a**,**b**).

In the proposed dual-direction scanning scheme, the transmission and reflection optical data are measured in the same cross section. Figure 4a,b shows the measurement for the top-to-bottom and top-to-top optical paths and three more top-to-bottom ones after a shift of bottom slab, respectively. To obtain such information at the same slice, especially for transmission, the opposite scanning slab is shifted by15 mm, the span of two arrays, along the Y direction in turn to complete the acquisition of 7S × 7D × 2 transmission data (Figure 4). As to the collection of reflection data, (4S × 3D + 3S × 4D) × 2 can be obtained in a single section through moving the top or bottom scanning slabs 15 mm in the Y direction. Furthermore, each of two scanning slabs can shift by 7.5 mm (half the span of two arrays) along the Y direction or the adjacent source and detection in one array along the X direction to accommodate the acquisition of optical data from a smaller breast or simply to acquire more data for the image reconstruction.

### 2.3. Calibration of Measurement System

The calibration of measurement system associated with an NIR DOT imaging system is a practical consideration. In our system, three important procedures are involved: (1) multichannel calibration, (2) PMT detector calibration, and (3) homogeneous phantom calibration. Multichannel calibration must consider the optical switches associated with the source fibers, LLGs, and scanning slabs in the system. The offset for each channel of the optical switch or each LLG was measured under fixed input power due to the channel difference of an optical switch or LLG differences in transmission. Because of probable alignment mismatches in the mechanical construction for detection on the top and bottom scanning slabs, the calibration was accomplished through each pair of excitation port and detection port between two plates, thereby establishing a set of correction factors for each source-and-detection pair. The second practical procedure is calibration for the response of two employed PMTs in their dynamic ranges. An adequate measurement range of the PMT for input light power under a user-selected gain setting was created to ensure the measurement was in the dynamic range; accordingly, the saturation point for the corresponding gain was found, and an ND filter operated by a preplanned program in LabVIEW was used in the measuring process to prevent the PMT from operating in a saturation range. The third consideration is the difference between data measured from the phantom and data calculated from the model; a standard homogenous phantom (*μ_a_* = 0.0103 mm^−1^ and *μ_s_’* = 0.91 mm^−1^ at 830 nm, E0823TA, ISS) was used.

## 3. Data Processing

In this data-processing section, the optical-property image reconstruction is summarized, and a feature index evaluation is proposed for identifying potential abnormal tissue. In NIR DOT imaging, the fundamental equation is modeled by the diffusion equation (DE)

(1) $$\nabla \cdot D(r)\nabla \Phi (r,\omega )-\left[\mu_{a}(r)-\frac{i\omega }{c_{n}}\right]\Phi (r,\omega )=-S(r,\omega )$$

where Φ(**r**, *ω*) denotes the photon fluence rate at position **r**, *ω* the light intensity modulation frequency, *S*(**r**, *ω*) the isotropic source term, *c_n_* (i.e., *c*/*n*, c and n are the speed of light in vacuum and the refractive index of the medium, respectively) the speed of light in tissue, and *μ_a_*(**r**) and *D*(**r**) are the optical absorption and diffusion coefficients, respectively. It governs light propagation in high-scattering biological tissues. Mappings of the absorption or scattering coefficients can be evaluated through the DE approximation based on the finite-element method (FEM). To solve Equation (1), the FEM based on the Galerkin weak form of Equation (1) along with a boundary condition, $-D\nabla \Phi \cdot \hat{n}=\alpha \Phi$ (flux, in fact), can be implemented. Thus, the following discrete equations in matrix form

(2) $$\underset{\mathrm{optical}-\mathrm{property}\;\mathrm{matrix}}{\underset{︸}{\left[\begin{array}{cc} A_{ij}^{bb}-\alpha B_{ij}^{bb} & A_{ij}^{bI}\\ A_{ij}^{Ib} & A_{ij}^{II} \end{array}\right]}}\underset{\mathrm{fluence}\;\mathrm{rate}\;\mathrm{matrix}}{\underset{︸}{\left\{\begin{array}{c} \Phi_{j}^{b}\\ \Phi_{j}^{I} \end{array}\right\}}}=\underset{\mathrm{source}\;\mathrm{matrix}}{\underset{︸}{\left\{\begin{array}{c} C_{i}^{b}\\ C_{i}^{I} \end{array}\right\}}}$$

can be obtained, where *A*, *B*, and *C* represent the total absorption and scattering effects in tissue, the boundary conditions, and the source term, respectively [25,26]. The forward solution, Φ, can be evaluated through Equation (2). Regarding the physical process, the fluence rate matrix Φ is quantitatively and qualitatively dependent on the source matrix *C* and the optical-property matrix *A*, respectively, where the optical-property matrix *A* is the inertia of the material despite being related to the wavelength. For simplicity, Equation (2) can be expressed as

(3) AΦ=C.

Subsequently, partially differentiating Equation (3) with ∂/∂μ_a_ and ∂/∂D, respectively, yields

(4) $$\Phi^{\prime }=-A^{-1}A^{\prime }\Phi +A^{-1}C^{\prime }.$$

With an approximation to applying the Taylor expansion method and ignoring higher order terms, we obtain

**Figure 5:**
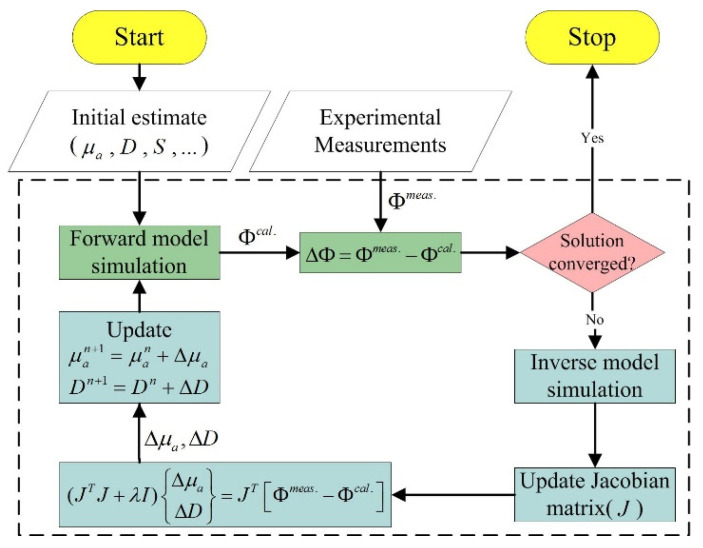
Flowchart of optical-property image reconstruction algorithm.

(5) JΔχ=ΔΦ,

where the Jacobian matrix *J* denotes the matrix consisting of *∂*Φ/*∂μ_a_* and *∂*Φ/*∂D*, Δχ denotes the vector composed of Δ*μ_a_* and Δ*D*, and ΔΦ denotes the vector with differences between the calculated photon fluence rate ($\Phi^{\mathrm{Cal}.}$) and measured photon fluence rate ($\Phi^{\mathrm{Meas}.}$). The elements of the Jacobian matrix can be calculated from Equation (4). Some high-performance inverse techniques [27,28] can be used for the data processing such as truncated singular value decomposition or genetic algorithms. Here, Tikhonov regularization was adopted to iteratively update the diffusion and absorption coefficients:

(6) $$\left[\begin{array}{c} \Delta \mu_{a}\\ \Delta D \end{array}\right]={\left(J^{T}J+\lambda I\right)}^{-1}J^{T}\left[\Phi^{\mathrm{Meas}.}-\Phi^{\mathrm{Cal}.}\right]$$

where λ is a regularization parameter to stabilize the solution. As described, this inversion generally requires the construction of the Jacobian matrix. The flowchart of the optical-property image reconstruction algorithm is shown in Figure 5. An in-house-coded FEM-based reconstruction program named DOpIm [29] that was updated from its previous graphical user interface program NIR·DOT_PC [24] has been developed using MATLAB^®^, in which the adjoint method [30,31] was used to construct the Jacobian efficiently and thus accelerate the evaluation of Equation (6). Furthermore, a rapid convergence method [32], edge-preserving regularization [33,34,35], and a guide to properly selecting multiple single-wavelength laser diodes [36] have been considered and implemented in the computation program.

**Figure 6:**
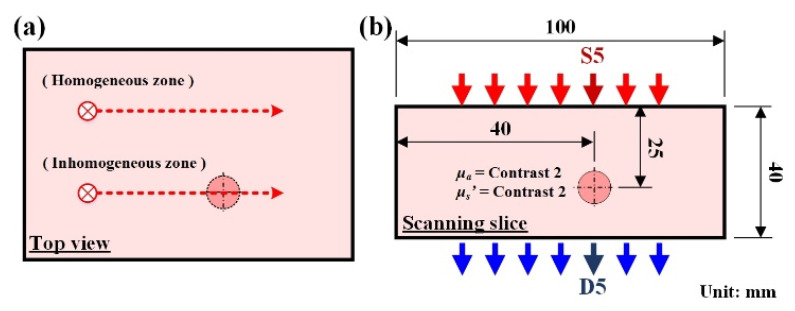
Inhomogeneous phantom schematic for illustrating feature index evaluation: (**a**) top view; (**b**) dimensions and optical property for the slice through an inhomogeneous zone.

**Figure 7:**
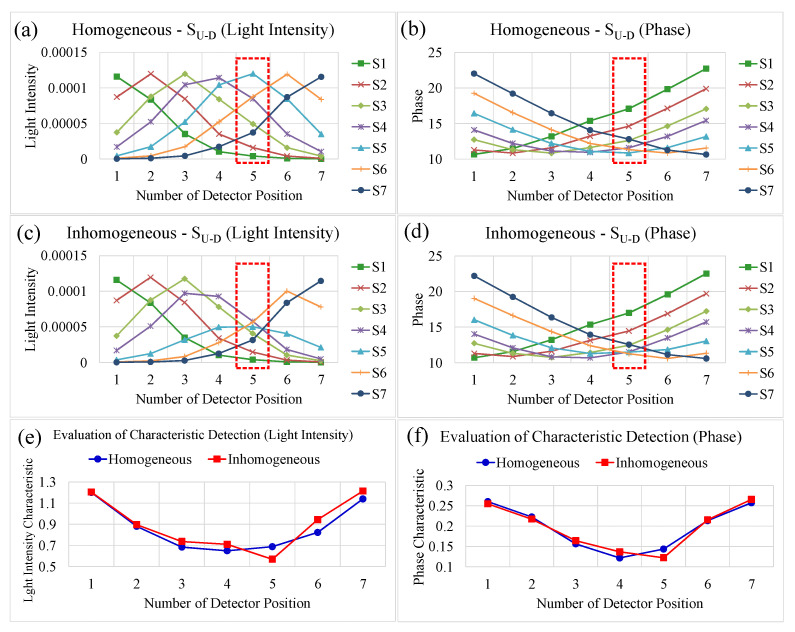
Examples illustrating the evaluation method: (**a**–**d**) present the intensity (W/mm^2^) and phase (°) outcomes of the homogenous and inhomogeneous slices, respectively, and (**e**,**f**) present the feature index curves for intensity and phase.

Generally, a total number of 2*N_s_* curves indicate the measured intensities and phases, *N_s_* curves for each, where *N_s_* is the number of provided sources that are required to show the measurement outcomes for a phantom/tissue; however, the acquired optical data curves are always indirect to the tumor existence except the level of overall absorption and scattering distribution. Here, we propose a feature index evaluation for the transmission measurement of each light-source position; therefore, one summarized curve that combines the data of *N_s_* curves is calculated to represent the feature of the test phantom or tissue, which is composed of characteristic indices at all detection positions. The feature index (χ) for a single detection is defined as

(7) $$\chi =\frac{\sigma_{D}}{{\bar{x}}_{D}}=\frac{\sqrt{\frac{\sum {x_{D}}^{2}-{\left(\sum x_{D}\right)}^{2}/N_{S}}{N_{S}-1}}}{{\bar{x}}_{D}},$$

where $x_{D}$ denotes the intensity (or phase) information of detection light, and ${\bar{x}}_{D}$ and $\sigma_{D}$ are the mean value and the standard deviation of the detection, respectively. As known, the malignant tissue possesses higher absorption due to the larger total hemoglobin concentration of angiogenesis and hyper-metabolism, and thus it is expected the feature indices for intensity may be evidently separate. Furthermore, while higher cellular density and fibrous tissue in tumor has lower oxygen saturation [37,38,39,40], the scattering phenomena surrounding malignant tissue may differ from normal tissue such that the phase difference feature changes. For further interpreting the significance of the feature index, the following example demonstrates this evaluation method. Figure 6a illustrates the scheme of light source scanning above the phantom (each for homogeneous and inhomogeneous zones) and the collection of optical data over the inhomogeneous slice (Figure 6b). For the fabricated phantom, its background has the optical properties of *μ_a_* = 0.0074 mm^−1^ and *μ_s_’* = 0.85 mm^−1^, and the embedded inclusion with 10 mm in diameter has twice the contrast of *μ*_a_ and *μ*_s_’. The measurement of intensity and phase is shown in Figure 7a,b for the homogeneous slice and Figure 7c,d for the inhomogeneous one. Moreover, Figure 7e,f presents the evaluation of feature index; these charts indicate that the inclusion can be evidently characterized at detection 5 (D5) with a dip, whereas it exhibits a U-shaped curve for the case of a homogeneous phantom. As can be seen, the feature index curves of intensity and phase can succinctly demonstrate the corresponding transmission measurements and illustrate useful information for the zone of abnormality.

## 4. Results and Evaluation

A pilot study here was conducted to reconstruct optical-property *μ_a_* and *μ_s_*’ images of the female breast, obtained by using NIR DOT in the FD plus model-based image reconstruction methods. We completed a series of optical breast examinations in five females (age range, 47–73 years); the protocol was approved by the Institutional Review Board of Taoyuan General Hospital (TYGH), and informed consent was obtained from the patients. The experimental protocol was as follows: The subject’s breast was compressed in the CC orientation parallel to the XOY plane (see Figure 3 for the reference coordinate system) or in the mediolateral oblique (MLO) view by using a standard mammographic level of compression. Subsequently, an X-ray tomosynthesis image of the breast was taken; the optical probe was set on its casing, altering the breast position as little as possible, and optical data were acquired for approximately 1.5 min per slice. A mesh consisting of 729 nodes and 1362 elements on average was used to represent the varied physical dimensions of the reconstruction domain. For each reconstruction case, a time of approximately 5 min was required on a computer with a 2.5 GHz CPU and 8 GB RAM. For suspected cancer cases, the biopsy examination was performed after both mammography and DOI.

A total of 10 mammographic data acquisitions on the CC and MLO views were performed on two right and three left breasts of the five volunteers. With the aid of mammograms that help physicians indicate suspect regions, the corresponding diffuse optical imaging through the proposed dual-direction measuring scheme was subsequently applied. Furthermore, the breast tissue from case 4 subject undergoing a mastectomy was imaged via DOI along three sections with tumors indicated by a mammogram. These examined breasts were compressed 30–60 mm; in detail, the physical dimension and source/detection positions are depicted in the following figures. Ringing artifacts along the boundary of the reconstructed optical-property images were ignored during inspection. We present five representative cases: a control measurement in one subject with no disease, one benign case, one suspected case, one invasive ductal carcinoma, and one positive case. Further, for all the suspected cases, a biopsy examination was performed after both mammography and DOI.

In this pilot clinical evaluation of the optical imaging system, we obtained two types of images, absorption and scattering, from a single slice. Physically, these represent two different intrinsic properties of breast tissue. For the absorption images, the breast masses demonstrated increased absorption relative to the background tissue; this increase in absorption can be primarily engendered by the increased blood volume associated with a rapidly growing tumor.

### 4.1. Case 1: Negative (BI-RADS 1)

**Figure 8:**
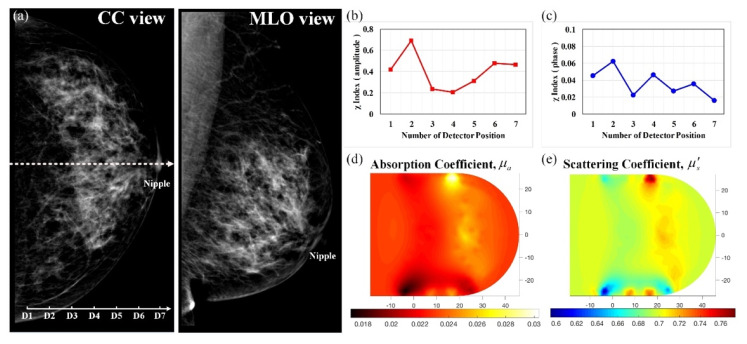
Healthy left breast of a 55-year-old female; (**a**) CC and MLO mammograms (the scale in 10 mm, the same hereafter for next mammograms); feature index curves of the (**b**) intensity and (**c**) phase; computed (**d**) *μ_a_* (mm^−1^) and (**e**) *μ_s_*’ (mm^−1^) images of the scanned slice along the dotted line of the CC mammogram.

A 55-year-old woman in menopause was under regular tracking for her left breast after her right breast had been removed because of the presence of masses. Figure 8 demonstrates optical images derived from this control subject with normal mammograms; Figure 8a displays the corresponding mammograms on CC and MLO views, showing that the left breast had moderately dense mammary tissue without any definite calcification. Moreover, Figure 8b,c shows the feature index curves of the measured intensity and phase, respectively, indicating U-shaped and oscillating curves, thus implying that the breast tissue with lacteal glands and fat had high absorption and scattering structures. In addition, Figure 8d,e show the reconstructed *μ_a_* and *μ_s_*’ images, respectively, displaying a broad range of slightly high contrast (approximately 0.028/0.024 for *μ_a_*) and average contrast (nearly 0.70 mm^−1^ for *μ_s_*’), matching with the dense mammary tissue in X-ray films.

### 4.2. Case 2: Benign (BI-RADS 2)

**Figure 9:**
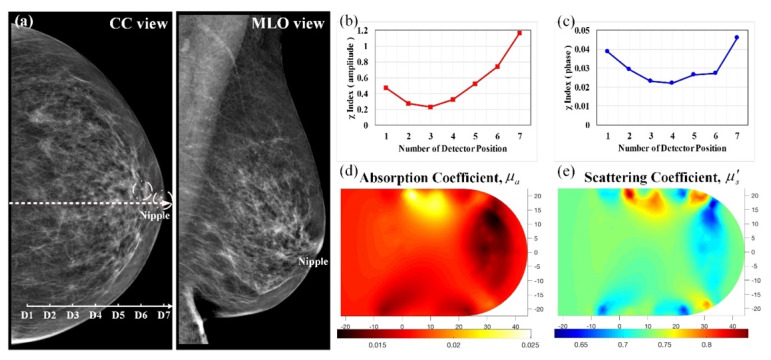
Left benign breast of a 73-year-old female; (**a**) CC and MLO mammograms, where dotted circles indicate nonspecific LNs; feature index curves of the (**b**) intensity and (**c**) phase; computed (**d**) *μ_a_* (mm^−1^) and (**e**) *μ_s_*’ (mm^−1^) images of the scanned slice along the dotted line of CC mammogram.

Figure 9 presents mammograms from the CC and MLO views and optical absorption and scattering images obtained from a 73-year-old woman under a routine breast cancer screening. Both of her breasts were found to be prominently fatty with little dense fibroglandular tissue and no definite focal mass lesion. Figure 9a shows focal pleomorphic calcifications in the subareolar region of her left breast and nonspecific lymph nodes (LNs). Moreover, Figure 9b,c shows U-shaped curves, implying that the breast tissue had a homogenous structure. In Figure 9d,e, the *μ_a_* and *μ_s_*’ images at the imaging plane are homogeneous in absorption and scattering in the scanned region, except for apparent ringing artifacts along the boundary. This case was eventually judged through biopsy examination as benign.

### 4.3. Case 3: Benign (BI-RADS 2)

**Figure 10:**
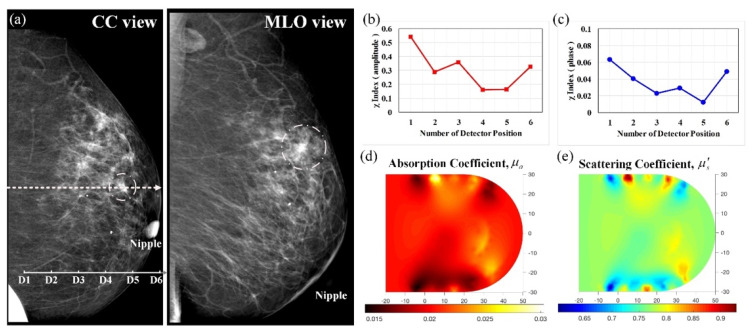
Suspected abnormality in the right breast of a 60-year-old female; (**a**) CC and MLO mammograms, where dotted circles indicate suspiciously ill-defined mass lesions; feature index curves of the (**b**) intensity and (**c**) phase; computed (**d**) *μ_a_* (mm^−1^) and (**e**) *μ_s_*’ (mm^−1^) images of the scanned slice along the dotted line of CC mammogram.

Figure 10 presents mammograms as well as optical absorption and scattering images of the right breast of a 60-year-old woman. This subject was also scanned using optical imaging, as shown in the optical-property images. Both of her breasts were prominently fatty with little dense fibroglandular tissue. Further, judging from the CC and MLO views, this study determined that the right breast had asymmetrically prominent dense tissue in the upper and outer portions, where the area had ill-defined mass lesions (Figure 10a). Subsequently, she was subjected to palpation that revealed a 10 × 10 mm^2^ area of focal mass. Figure 10b,c shows the curves indicating characteristics of lower values around D4 to D5. However, the reconstructed *μ_a_* and *μ_s_*’ images depicted in Figure 10d,e do not show the area as a higher contrast after the optical imaging; the average levels were approximately 0.020 and 0.78 mm^−1^ for *μ_a_* and *μ_s_*’, respectively. Again, this case was judged as benign through biopsy examination.

### 4.4. Case 4: Positive with Invasive Ductal Carcinoma (BI-RADS 4)

**Figure 11:**
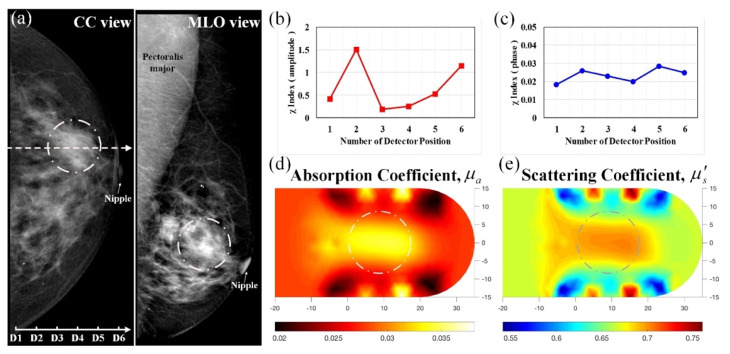
Infiltrating ductal carcinoma in the right breast of a 59-year-old female; (**a**) CC and MLO mammograms; feature index curves of the (**b**) intensity and (**c**) phase; computed (**d**) *μ_a_* (mm^−1^) and (**e**) *μ_s_*’ (mm^−1^) images of the scanned slice along the dotted line of CC mammogram.

Figure 11 depicts mammograms as well as optical absorption and scattering images derived from a 59-year-old female with a palpable mass in the right breast. The biopsy examination indicated that she had an infiltrating ductal carcinoma of large distribution. Figure 11a shows mammographic images of CC and MLO views, revealing obscure, oval, and microlobulated masses 5.3 cm away from the nipple at the right outer lower quadrant; further surgical excision and pathologic examination were recommended. The feature index curves in Figure 11b,c indicate obvious lower values around D3 to D5. Figure 11d shows the reconstructed *μ_a_* image as a craniocaudal slice, exhibiting a marked absorption increase (nearly 0.035/0.025) at the location corresponding to the tumor region that appears in the mammogram. The *μ_s_’* image in Figure 11e shows the distribution at 0.6–0.7 mm^−1^, also displaying a marked increase in the tumor region.

**Figure 12:**
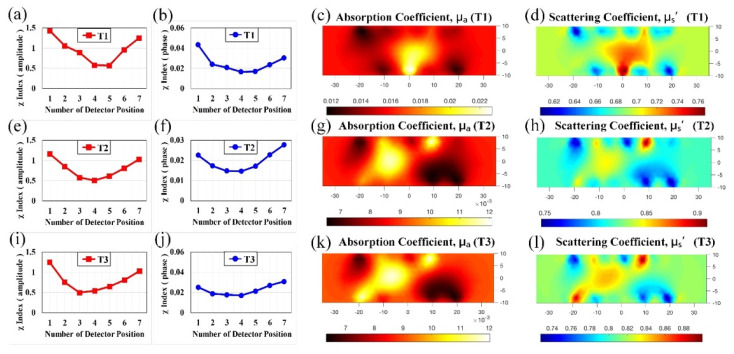
Feature index curves of the intensity and phase, and computed *μ_a_* (mm^−1^) and *μ_s_*’ (mm^−1^) images, of the scanned slice along T1 (**a**–**d**), T2 (**e**–**h**), and T3 (**i**–**l**).

As mentioned, representative slices were taken and labeled as T1, T2, and T3, each of which scans from the lower to the upper ‘×’ marks. In addition to the reconstructed absorption and scattering images scanned along T1–T3, the corresponding feature index curves of the intensity and phase were plotted, as shown in Figure 12a,b, Figure 12e,f, Figure 12i,j, respectively. As illustrated in Figure 12a,b,e,f,i,j, the features occurred at the lower portion of feature index curves, i.e., the regions near D4–D5, D4, and D3–D4 (D4 denotes detector position #4, and vice versa), respectively, for T1, T2, and T3; posterior to the corresponding reconstruction, the reconstructed optical-property images demonstrate areas with higher contrast (approximately 1.37), indicating the mass positions as well. The details of the quantities are provided in Table 1. It should be noted that for each section of T1, T2, and T3 the optical data acquired ranges between two ‘×’ marks (Figure S1c) around 90 mm long, but the reconstructed images were performed and presented for the inner 70 mm (Figure 12).

**Table 1:** Average values of optical properties of the background and tumor tissues for cases 1–5.

	Background		Tumor		Ratio		Area (%)	
	$\mu_{a}$(mm^−1^)	${\mu^{\prime }}_{s}$(mm^−1^)	$\mu_{a}$(mm^−1^)	${\mu^{\prime }}_{s}$(mm^−1^)	$\mu_{a}$	${\mu^{\prime }}_{s}$	$\mu_{a}$	${\mu^{\prime }}_{s}$
Figure 8	0.0236	0.698	NA	NA	NA	NA	NA	NA
Figure 9	0.0174	0.728	NA	NA	NA	NA	NA	NA
Figure 10	0.0202	0.785	NA	NA	NA	NA	NA	NA
Figure 11	0.0252	0.647	0.0345	0.703	1.37	1.09	22.60	30.53
Figure 12 (T1)	0.0154	0.674	0.0215	0.729	1.40	1.08	10.12	17.66
Figure 12 (T2)	0.0087	0.810	0.0117	0.847	1.35	1.05	14.88	14.68
Figure 12 (T3)	0.0085	0.806	0.0118	0.845	1.39	1.05	15.48	14.09
Figure 13	0.0159	1.126	0.0191	1.184	1.20	1.05	24.57	25.24

NA = not applicable.

### 4.5. Case 5: Positive (BI-RADS 6)

**Figure 13:**
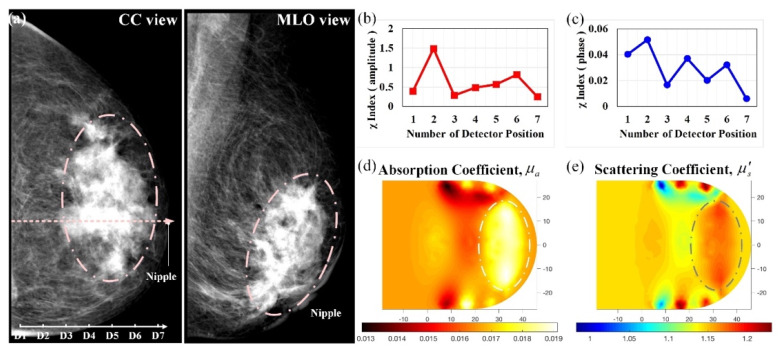
Abnormality in the left breast of a 47-year-old female; (**a**) CC and MLO mammograms; feature index curves of the (**b**) intensity and (**c**) phase; computed (**d**) *μ_a_* (mm^−1^) and (**e**) *μ_s_*’ (mm^−1^) images of the scanned slice along the dotted line of CC mammogram.

Figure 13 shows mammograms as well as optical absorption and scattering images from the left breast of a 47-year-old woman. Her left breast exhibited retraction around the nipple and adjacent skin; an X-ray mammography process was promptly performed. Figure 13a shows the heterogeneously dense breast, with an asymmetrical structure of distortion and dense mammary tissue, in which obscured irregular dense masses could be observed in the subareolar region; malignant masses measuring 80 × 47 mm^2^ were identified, and this was proven with biopsy. The subject was also investigated using our optical imaging system, and the investigation results are shown using feature index curves and optical-property images in Figure 13b–e. The curves display a suspicious area between D3 and D7, which was associated with low and oscillating values as shown (Figure 13b,c). Further, the corresponding optical-property images show a bulky bright area.

These clinical cases demonstrate and preliminarily verify the developed scanning system. The computed optical property seemed to be underestimated according to the past published reports [3,6,7,8]; thus, the contrasts in practice are higher than the currently estimated values. The tumor was found near the boundary in the subject diagnosed as benign (Case 2); however, it was hidden under the image artifacts in our reconstruction. Moreover, the ringing artifacts along the boundary may cover the existing mass; this requires further study and improvement on the imaging schemes.

Table 1 provides the average optical properties for both tumor and background regions of cases 1–5. As computed, the average absorption and scattering properties were in the range 0.017 ± 0.008 and 0.88 ± 0.24 mm^−1^ for *μ_a_* and *μ_s_*’ of the background, respectively, and 0.023 ± 0.012 and 0.95 ± 0.25 mm^−1^ for *μ_a_* and *μ_s_*’ of the mass. Because an obvious contrast was presented in the absorption images (Figure 8, Figure 9, Figure 10, Figure 11, Figure 12, Figure 13), the tumor-to-background ratio was 1.20–1.40; in practice, the contrast ratio should be higher owing to the underestimation in calculation. In case 5 (Figure 13), the mass was large enough (approximately 25%) to be detected despite the low contrast (nearly 1.20). For further comparison, absorption of T1, T2, and T3 in the specimen is lower, probably due to the blood exudation out of tissues. As illustrated in Figure 8, Figure 9, Figure 10, Figure 11, Figure 12, Figure 13 and Table 1, our developed DOI system has the potential for breast tumor or lesion screening.

## 5. Conclusions

We provide in vivo experimental evidence for breast screening, as derived from a pilot study in Taiwan, of a self-developed NIR DOI system. From FD tomographic measurements, both *μ_a_* and *μ_s_*’ images can be reconstructed to show abnormal findings when tumors were present. The proposed feature index curves of intensity and phase can succinctly demonstrate the corresponding transmission measurements; further, the evaluated feature indices may provide an insight into the existence of probably malignant tissues. In case 3, the FD DOI system enabled the suspected tumor to be differentiated as benign (with low contrast to the background); this may be attributed to the higher sensitivity of optical imaging in the absorption of abnormalities. The presented scattering images exposed the structure densities, but revealed no abnormalities in the cases. However, ringing artifacts were observed around the boundaries; this might be due to the limited acquired information or the mesh used.

In particular, an ROI analysis demonstrated significant differences in absorption between healthy and malignant breasts, in which an average contrast between abnormalities and background tissue beyond 1.4 could be found. Except for ringing artifacts, the average scattering property of the structure densities was approximately 0.85 mm^−1^. With respect to mammograms, this study also showed that the NIR DOI system can probe the difference between breast tumors and normal tissue, which is useful as a contrast mechanism for breast screening. As aforementioned in Section 2.1, the developed measuring module takes about 90 s to acquire opto-electrical data for one slice of image to be reconstructed. It has no radio-hazard concerns related to the measurement due to employing NIR light sources without ionizing radiation, though the module was incorporated with a mammography machine. At the current stage, the breast was squeezed to 30 to 60 mm thick between two working plates during examination (Figure 8d,e, Figure 9d,e, Figure 10d,e, Figure 11d,e and Figure 13d,e).

In the future, we plan to design a series of blind in vivo tests to verify this developed NIR DOI system for breast tumor screening. Moreover, our next development aims to study the system performance for breasts with lesions under ring-based scanning without compression, because this is obviously important for proving the clinical significance of the DOI system.

## 6. Patents

1. Pan, M.-Chun, Chiang, H.-Ch., Chen, Ch.-Y., Chen, L.-Y., Wu, Ch.-T., Pan, M.-Cheng (2012). U.S. Patent No. 8,378,302B2. Washington, DC: U.S. Patent and Trademark Office.
2. Pan, M.-Chun, Yu, J.-M., Chiang, H.-Ch., Pan, M.-Cheng, Chen, Ch.-Y., Chen, L.-Y. (2013). U.S. Patent No. 8,395,120B2. Washington, DC: U.S. Patent and Trademark Office.
3. Pan, M.-Chun, Yu, J.-M., Chiang, H.-Ch., Pan, M.-Cheng, Chen, Ch.-Y., Chen, L.-Y. (2014). R.O.C. Patent No. I461179. Taipei: R.O.C. Patent and Trademark Office.
4. Pan, M.-Chun, Chiang, H.-Ch., Chen, Li.-Y., Pan, M.-Ch. (2014) US patent No. 8,712,136B2. Washington, DC: U.S. Patent and Trademark Office.
5. Pan, M.-Chun, Chiang, H.-Ch., Chen, Li.-Y., Pan, M.-Ch. (2014) R.O.C. patent No. I433051. Taipei: R.O.C. Patent and Trademark Office.

## Supplementary Materials

The following supporting information can be downloaded at: https://www.mdpi.com/article/10.3390/biomedicines10051040/s1, Figure S1: Further investigation on breast tissues from case 4 subject undergoing a mastectomy.
