# Supplementary material for: In Vivo Validation of Diffuse Optical Imaging with a Dual-Direction Measuring Module of Parallel-Plate Architecture for Breast Tumor Detection
Source: Biomedicines. 2022 Apr 30;10(5):1040. doi: 10.3390/biomedicines10051040 (PMC9138400; doi:10.3390/biomedicines10051040)
Supplement: Supplementary file 1 [file biomedicines-10-01040-s001.zip › biomedicines-1669979-supplementary.pdf]

biomedicines

After the mammography and NIR DOI processes, she underwent a modified radical mastectomy (MRM) and pathologic examination, which showed invasive ductal carcinoma [pT2(m)N0] in the right breast. Furthermore, the specimen submitted in two bottles consisted of an MRM specimen and axillary LNs fixed in formalin. Correspondingly, the specimen was investigated using both mammography and DOI. Figures S1(a) and S1(b) present optical-data acquired from the tissues and the subsequent resulting mammogram, respectively; Fig. S1(c) illustrates a schematic of masses in the specimen, with the slices labeled T1, T2, and T3 for scanning in.

The MRM specimen measuring 19 × 17 × 4 cm^3 was yellowish and soft, and it contained an ellipse of skin (12 × 4 cm^2) with a nipple. A pathologic check revealed five tumors scattered in the breast, measuring from 0.5 × 0.5 × 0.4 to 3 × 2.5 × 1.8 cm^3 and located at 1–9 o'clock 2–5.5 cm away from the nipple. The other specimen consisted of two tissue fragments measuring 3.5 × 2 × 1 to 5 × 4 × 2 cm^3; several LNs were dissected out, the largest of which was 1 cm.

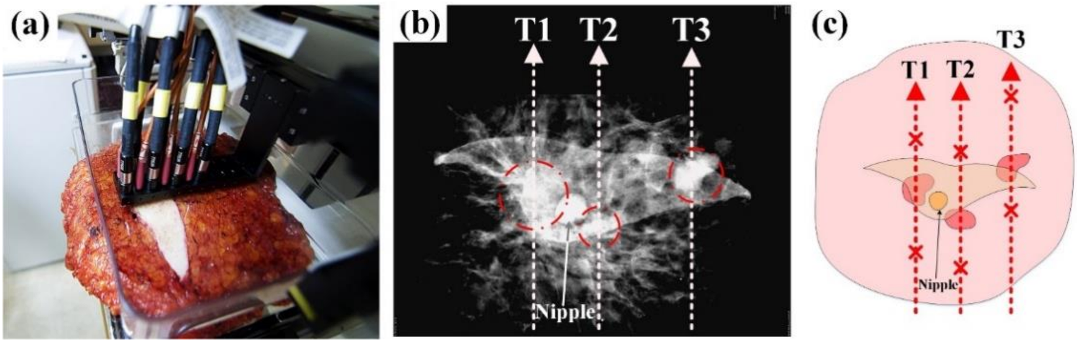

Figure S1. Further investigation on breast tissues from case-4 subject undergoing a mastectomy, (a) tissues ready to be optical-data acquired, (b) mammogram of the tissues, and (c) schematic diagram of masses in the tissues.
